# Genomic architecture of autism spectrum disorder in Qatar: The BARAKA-Qatar Study

**DOI:** 10.1186/s13073-023-01228-w

**Published:** 2023-10-07

**Authors:** Mona Abdi, Elbay Aliyev, Brett Trost, Muhammad Kohailan, Waleed Aamer, Najeeb Syed, Rulan Shaath, Geethanjali Devadoss Gandhi, Worrawat Engchuan, Jennifer Howe, Bhooma Thiruvahindrapuram, Melissa Geng, Joe Whitney, Amira Syed, Jyothi Lakshmi, Sura Hussein, Najwa Albashir, Amal Hussein, Ilaria Poggiolini, Saba F. Elhag, Sasirekha Palaniswamy, Marios Kambouris, Maria de Fatima Janjua, Mohamed O. El Tahir, Ahsan Nazeer, Durre Shahwar, Muhammad Waqar Azeem, Younes Mokrab, Nazim Abdel Aati, Ammira Akil, Stephen W. Scherer, Madeeha Kamal, Khalid A. Fakhro

**Affiliations:** 1 College of Health and Life Sciences, Hamad Bin Khalifa University, Doha, Qatar; 2 Department of Genetics, Sidra Medicine, Doha, Qatar; 3 The Centre for Applied Genomics, The Hospital for Sick Children, Toronto, ON Canada; 4 Genetics and Genome Biology Program, The Hospital for Sick Children, Toronto, ON Canada; 5 Genomics Data Science Core, Sidra Medicine, Doha, Qatar; 6 Department of Molecular Genetics, University of Toronto, Toronto, ON Canada; 7 Hamad Medical Corporation, Doha, Qatar; 8 Pathology and Laboratory Medicine Department, Genetics Division, Sidra Medicine, Doha, Qatar; 9 Department of Pediatrics, Sidra Medicine, Doha, Qatar; 10 Department of Psychiatry, Sidra Medicine, Doha, Qatar; 11 Weill Cornell Medicine, Doha, Qatar; 12 Department of Genetic Medicine, Weill Cornell Medicine, Doha, Qatar; 13 Qatar University, Doha, Qatar; 14 McLaughlin Centre, University of Toronto, Toronto, ON Canada

**Keywords:** Autism spectrum disorder, ASD, BARAKA cohort, ASD risk genes, De novo variants, Whole genome sequencing, SNVs, Middle Eastern population

## Abstract

**Background:**

Autism spectrum disorder (ASD) is a neurodevelopmental condition characterized by impaired social and communication skills, restricted interests, and repetitive behaviors. The prevalence of ASD among children in Qatar was recently estimated to be 1.1%, though the genetic architecture underlying ASD both in Qatar and the greater Middle East has been largely unexplored. Here, we describe the first genomic data release from the BARAKA-Qatar Study—a nationwide program building a broadly consented biorepository of individuals with ASD and their families available for sample and data sharing and multi-omics research.

**Methods:**

In this first release, we present a comprehensive analysis of whole-genome sequencing (WGS) data of the first 100 families (372 individuals), investigating the genetic architecture, including single-nucleotide variants (SNVs), copy number variants (CNVs), tandem repeat expansions (TREs), as well as mitochondrial DNA variants (mtDNA) segregating with ASD in local families.

**Results:**

Overall, we identify potentially pathogenic variants in known genes or regions in 27 out of 100 families (27%), of which 11 variants (40.7%) were classified as pathogenic or likely-pathogenic based on American College of Medical Genetics (ACMG) guidelines. Dominant variants, including de novo and inherited, contributed to 15 (55.6%) of these families, consisting of SNVs/indels (66.7%), CNVs (13.3%), TREs (13.3%), and mtDNA variants (6.7%). Moreover, homozygous variants were found in 7 families (25.9%), with a sixfold increase in homozygous burden in consanguineous versus non-consanguineous families (13.6% and 1.8%, respectively). Furthermore, 28 novel ASD candidate genes were identified in 20 families, 23 of which had recurrent hits in MSSNG and SSC cohorts.

**Conclusions:**

This study illustrates the value of ASD studies in under-represented populations and the importance of WGS as a comprehensive tool for establishing a molecular diagnosis for families with ASD. Moreover, it uncovers a significant role for recessive variation in ASD architecture in consanguineous settings and provides a unique resource of Middle Eastern genomes for future research to the global ASD community.

**Supplementary Information:**

The online version contains supplementary material available at 10.1186/s13073-023-01228-w.

## Background

Autism spectrum disorder (ASD) is a neurodevelopmental condition characterized by impaired social interactions, deficits in communication, restricted interests, and repetitive behaviors [1]. ASD often co-occurs with other conditions, including intellectual disability (ID), attention-deficit hyperactivity disorder (ADHD), epilepsy, and gastrointestinal (GI) problems [2]. Various factors, including genetic, epigenetic, environment, and hormonal changes contribute to the broad phenotypic spectrum of ASD. The high heritability of ASD (70–90% based on twin studies) [3] and the increased relative risk to siblings (10–20-fold) suggest that genetic factors play a prominent role in ASD etiology [4].

Advances in both genomic technologies and ASD phenotyping have improved our understanding of the genetic architecture of ASD. Studies of genomic data at scale have revealed over hundreds genes and variants to be associated with ASD, disrupting key biological processes such as neurotransmission, synapse function, chromatin remodeling, cortical development, and metabolism [4, 5].

De novo variation in coding regions, including SNVs, small insertions or deletions (indels), and structural variants (SVs), together account for 10–30% of simplex ASD cases [2, 6, 7]. Recently, other variant classes such as TREs and mitochondrial variants have been shown to contribute to ASD susceptibility in large population cohorts [8–10]. Furthermore, the use of statistical methods such as the transmission and de novo association analysis (TADA) helped identify risk genes by combining both de novo and transmitted SNVs/Indels [11]. A recent study applied TADA analysis and highlighted 134 dominant genes to be ASD-associated with false discovery rate < 0.1 [12].

There has been growing evidence implicating recessive variation in ASD susceptibility, especially in consanguineous settings (approximately 5% of all ASD cases) [13, 14]. Rare homozygous loss-of-function (LoF) variants have been described in several genes such as *CA2, DDHD1, FEV, NSUN2, PAH, SLC1A1,* and *USH2A* [15, 16]. Despite these discoveries, recessive causes of ASD generally form a minority of the overall genetic architecture of ASD among large cohorts published to date, estimated at around 1.1% in MSSNG and 0.3% in the SSC datasets [10]. Additionally, recent studies that focused on families with high consanguinity have demonstrated a higher rate of recessive causes, e.g., 39% [17], suggesting the recessive burden in ASD is yet to be explored among global consanguineous populations.

Successful molecular diagnosis of individuals with ASD brings several benefits allowing earlier behavioral interventions, assessment of familial recurrence risk (low in case of de novo mutation) as well as informing more precise interventions. Nevertheless, despite the improvements in understanding the genetics of ASD, most discoveries have been only produced in certain geographical areas, which limits the diversity of ethnic backgrounds that can benefit from research. In ASD research, for instance, people of non-European ancestry are still significantly underrepresented [10], with those of Middle Eastern origin being among the most underrepresented globally.

ASD research has recently received a lot of attention in Qatar. The incidence of ASD in Qatar is estimated to be 1 in 87 (1.1%) [18], which is relatively similar to the global estimates in different populations [19, 20]; however, the genetic architecture of ASD in Arab world remains poorly explored. The BARAKA study (Building a Resource for the Advancement of Knowledge of Autism in Qatar) aims to establish a national resource on ASD research, consisting of a biorepository of samples and data on patients at Sidra Medicine broadly consented for research. The repository hosts extensive clinical and questionnaire data on each individual including electronic health records (EHR), aliquots of whole blood, plasma, cells, RNA, saliva, and microbiome samples. Importantly, most patients were consented to be recontacted in the future. This resource is expected to be a valuable resource contributing to regional and global efforts investigating genetic and environmental determinants of ASD.

Herein, we describe the results of BARAKA-WGS analysis of 100 families (372 subjects), where we comprehensively investigate the genetic architecture (including dominant/recessive, nuclear/mitochondrial variants) contributing to ASD. Being the first comprehensive genomic study of ASD from the Middle East, this sets an important baseline for understanding the architecture of this complex condition in highly consanguineous populations.

## Methods

### Cohort description and phenotyping

A total of 100 families (372 total individuals, including 104 individuals with ASD plus their parents and unaffected siblings) were enrolled from Sidra Medicine’s various pediatric clinics (Developmental Pediatrics, Child and Adolescent Psychiatry, Adolescent Medicine) as part of the BARAKA-Qatar study cohort. Most of the families where simplex (98/100) and only two families where multiplex families both with 3 affected siblings each. The majority of families were of Arab descent (58%), followed by South Asian (25%), European (7%), African (5%), and other ethnicities. Children with known karyotyping abnormalities, Fragile X syndrome, and Rett syndrome were excluded. ASD diagnosis was made following standard autism diagnostic measures (DSM-V). The study was approved by the institutional review board (IRB) of Sidra Medicine (IRB No. 1500767), and written informed consent was obtained from all participants (the full description of the cohort phenotypes is presented in Additional file 1: Table S1 and Additional file 2: Figure S1). De novo SNVs/SVs and compound heterozygous variants analysis were performed only on complete trios (79% of families).

### WGS and variant detection

Whole blood samples were collected from individuals with ASD and family members. Total genomic DNA was extracted using the DNeasy Blood & Tissue Kit (Qiagen sciences LLC, Germantown, MD, USA) according to the manufacturer’s instructions. DNA samples were processed at Sidra Medicine as previously described [21]. Briefly, samples were sequenced (150 bp paired-end reads) using Illumina HiSeq X to a minimum depth of 30 × , and reads were aligned to GRCh37/hg19 using BWA version 0.7.10 [22]. Sequence-level variants were detected with GATK version 3.3 using the best practices pipeline [23]. VCF files were annotated using the SnpEff/SnpSift tool [24] by adding allele frequencies from variant databases (1000 Genomes Project [25], gnomAD [26], and ExAC [27], and Qatar-genome project (QGP)). De novo variants were detected in complete trios (*n* = 79) using a combination of three tools (VarScan [28], RUFUS [29], and FreeBayes [30]) as previously described [31]. All variants reported in this study were lifted over to GRCh38/hg38 using Broad Institute liftover tool (https://liftover.broadinstitute.org) [32].

### SNV and indel analysis

#### Quality filtration

We retained variants that met all the following criteria: (i) flagged as “PASS” all GATK filters, (ii) genotype quality (GQ) ≥ 10, (iii) read depth ≥ 20, (iv) allele fraction between 0.2 and 0.8 (for heterozygous variants), and (v) not present in low-complexity regions. Rare variants were defined as those with minor allele frequencies (MAF) < 1% in all general databases such as 1000G, gnomAD, ExAC, QGP, and an internal database of > 35,000 alleles sequenced as part of various projects at Sidra Medicine. To determine the level of consanguinity from our cohort, we used KING for pair-wise measurement of relationships (–-kinship command, with a cutoff of ≥ 0.044) (Additional file 2: Figure S2) [33] and calculated inbreeding coefficient (F) for per-sample using plink1.9 (–het command with cutoff > 0.1) (Additional file 2: Figure S3) [34].

#### Variant prioritization

De novo, homozygous, compound heterozygous, and X-linked recessive variants that are rare and coding were considered to be potentially pathogenic if they met the following criteria: (i) LoF effect on the protein (stop gain, frameshift deletion, frameshift insertion, or canonical splice site variation) or (ii) damaging missense variants (Dmiss), defined as variants deemed deleterious by at least 5 in silico prediction tools. These tools included CADD (threshold for deleteriousness ≥ 10) [35], SIFT (deleterious) [36], PolyPhen2-HDIV (probably-damaging or possibly damaging) [37], PolyPhen2-HVAR (probably-damaging or possibly damaging) [37], LRT (deleterious) [38], MutationAssessor (high or medium) [39], MutationTaster (deleterious) [40], MPC score (≥ 1) [41], and PROVEAN (deleterious) [42].

Gene constraint was assessed using the gnomAD pLI score for dominant variants and pRec score for recessive variants. Variants were also screened for any phenotypic association in the database of Online Mendelian Inheritance in Man (OMIM) [43]. Variants found in genes causing phenotypes relevant to ASD (such as developmental delay (DD), intellectual disability, etc.) were curated based on American College of Medical Genetics (ACMG) guidelines [44] using Franklin and InterVar (Available online: https://franklin.genoox.com, [45]). (Note: For all de novo variants, PS2 criteria were manually adjusted).

#### Known ASD/NDD panel genes/regions

To further prioritize likely ASD-associated variants, we identified variants impacting genes in a list of known neurodevelopmental disorder (NDD)/ASD genes, which included the Genomics England NDD/autism panel genes and Simons Foundation Autism Research Initiative (SFARI) genes with a score of 1. This panel contained 1714 genes (634 dominant, 942 biallelic, and 138 X-linked; Additional file 1: Table S2). CNVs that overlap previously published list of genes/regions described as pathogenic to ASD [12] or known NDD/ASD genes were defined as “known” CNVs. In addition, we investigated TREs that affect known ASD genes from the recently reported list (57 genes) in ASD [8].

#### Novel genes/regions associated with ASD/NDD

In addition to identifying damaging variants in known genes, we flagged damaging de novo and rare homozygous variants (LoF, Dmiss) in novel candidate genes. For de novo variants, we leveraged other ASD cohorts (MSSNG, SSC, and SPARK) to look for additional individuals with evidence in these same genes. For homozygous variants, we used an additional filter of genes with high pRec scores (> 0.9). We also considered de novo or homozygous CNVs in novel genes/regions. In both cases, we also looked in other ASD cohorts for additional individuals with variants of the same category and inheritance patterns in the same gene to strengthen evidence for causality.

### CNV detection and analysis

CNV detection was performed using a pipeline comprising multiple algorithms: CNVnator [46], DELLY [47], ERDS [48], Manta [49], Speedseq [50], and SvABA [51]. We retained only CNVs detected by at least two tools to increase specificity. We then merged CNVs detected by the 6 tools if they were of the same type and their start and end coordinates were within 500 bp window. First we merged CNVs within each individual to generate a unique set of CNVs per-sample and subsequently across individuals to create a population-level variant file using Survivor (version 1.0.7) [52], which was then annotated using AnnotSV (version 2.2) [53]. De novo and homozygous CNVs were identified using custom scripts with the following additional allele frequency filters (allele frequency < 0.1% for de novo and < 1% for homozygous) from global biobank SVs studies [54–56]. After filtering, we visualized CNVs using samplot (version 1.0.17) [57].

### Variant validation

We selected 12 de novo variants to confirm using Sanger sequencing as previously described [58]. As a further quality check, we used digital-droplet PCR (ddPCR) to validate a subset of CNVs, as described previously [59]. We successfully confirmed all de novo SNVs and CNVs (Additional file 2: Figure S4) 

### Calling of tandem repeats and expansions

Genome-wide detection of tandem repeats expansions (TREs) was performed using ExpansionHunter Denovo (EHdn) [60], which uses anchored in-repeat reads to estimate the size and location of tandem repeats, using the same pipeline as previously described [8].

### Mitochondrial variant calling

Variant calling in mitochondrial DNA was performed using Mutect2 (GATK v4.1.2.0) [23] using the newly implemented –mitochondria option. We only kept properly mapped reads for variant calling and filtered these using the FilterMutectCalls options. Left alignment and trimming were performed on variants and only variants with the PASS filter were retained for further analysis.

## Results

### Cohort description

All individuals with ASD in the BARAKA Study met diagnostic criteria according to the Diagnostic and Statistical Manual of Mental Disorders (DSM-5) (American Psychiatric Association, 2013). A total of 104 affected individuals from 100 families (79% complete trios) were analyzed, including 98 simplex and 2 multiplex families (both with 3 affected siblings each), with a male to female ratio of 5.5 (88 males and 16 females). The most common comorbidities among the BARAKA cohort were ADHD (35.6%), ID (29.8%), DD (28.8%), GI problems (19.2%), learning disabilities (10.6%), and seizures (6.7%) (Table 1, Additional file 2: Figure S1). Consistent with the demographic breakdown of Qatar, the majority of families were of Arab descent (58%), followed by South Asian (25%), European (7%), African (5%), and other ethnicities. In total, 44 out of 100 families (44%) were consanguineous (Additional file 2: Figure S2 and Figure S3).

**Table 1:** Summary of cohort and associated comorbidities

	Number of individuals (%)
Sex	
Female	16 (15.4%)
Male	88 (84.6%)
Additional clinical comorbidities	
ADHD	37 (35.6%)
Intellectual disability	31 (29.8%)
Developmental delay—speech	
Verbal	96 (92.3%)
Non-verbal	8 (7.7%)
Developmental delay—motor	22 (21.1%)
Learning disabilities	11 (10.6%)
Seizures	7 (6.7%)
GI problem	20 (19.2%)

### WGS and variant discovery

**Fig. 1:**
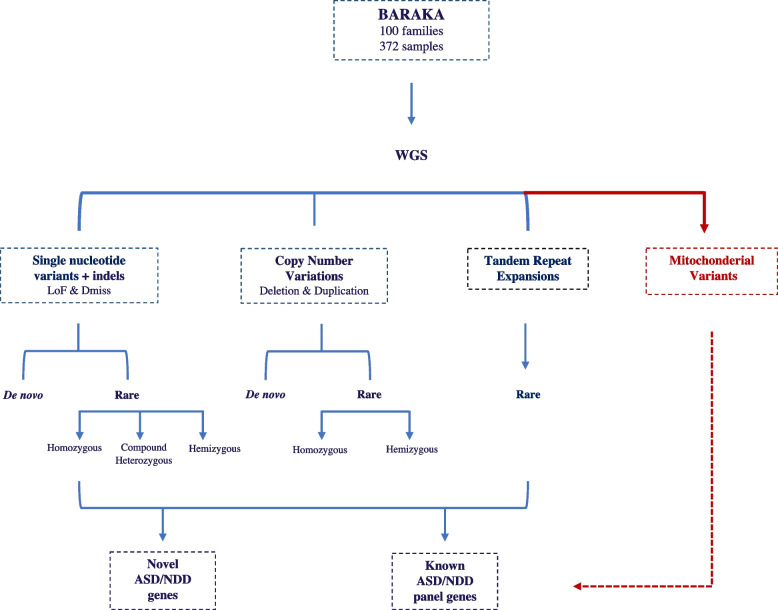
Overview of WGS approach and variant prioritization

All children and their families (*n* = 372 individuals) underwent WGS to an average read depth of 36 × ,while almost 96% of bases were covered at a mean depth of 20. Individuals had, on average, 4,206,499 SNVs and 110,600 indels per genome. After filtering variants based on MAF < 1% in general population databases such as 1000G, gnomAD, ExAC, and an extensive internal database of > 15,000 Qatari alleles, an average of 26,743 rare SNVs (95.3% heterozygous and 4.7% homozygous) and 67,292 rare indels (87.8% heterozygous and 12.2% homozygous) per genome remained for downstream analysis (Fig. 1). We then proceeded with a two-tier approach—first investigating variants of different classes in known ASD genes, and then transitioning genome-wide for putatively novel candidate genes causing ASD in this cohort.

### Pathogenic variants in known ASD-risk genes and regions

#### Small variants (SNVs + indels)

We first sought to identify (DN) or rare inherited LoF or Damaging missense (Dmiss) variants in 1714 known NDD and ASD genes (curated from multiple sources as described in “Methods”) and found 26 such variants in 24 genes in 24 individuals (Table 2). Nine families had nine DN variants in known ASD/NDD genes (*STAG1, SCN2A, MTOR, WDR37, EIF5A, KCNMA1, KDM5B GRIN2B,* and *MYO5A*). All of these variants were Dmiss except for one LoF in *KCNMA1.* Two variants (p.Arg373Gln in *STAG1* and p.Ala1773Val in *SCN2A*) were already reported as pathogenic in ClinVar for complex neurodevelopmental disorders. Using ACMG classification, the seven remaining DN variants were scored as likely pathogenic. One paternally inherited heterozygous variant (p.Arg266Cys) in *DNM1* was shared between three siblings with ASD and scored as VUS (Table 2).

**Table 2:** Candidate variants in known ASD/NDD genes/regions by variant type

**A. SNVs + indels (dominant)**										
**Proband_ID**	**Sex**	**Gene**	**Position (hg19)**	**Position (hg38)**	**Inheritance**	**Variant type**	**HGVS.c**	**HGVS.p**	**Relevant OMIM phenotypes**	**ACMG**
BRK-48–01	M	*WDR37*	chr10:1,126,397	chr10:1,080,457	De novo	Dmiss	c.377A > T	p.Tyr126Phe	Neurooculocardiogenitourinary syndrome (AD)	LP
BRK-52–01	F	*STAG1*	chr3:136,192,388	chr3:136,473,546	De novo	Dmiss	c.1118G > A	p.Arg373Gln	Intellectual developmental disorder (AD)	P
BRK-98–01	F	*SCN2A*	chr2:166,245,634	chr2:165,389,124	De novo	Dmiss	c.5318C > T	p.Ala1773Val	Developmental and epileptic encephalopathy (AD)	P
BRK-78–01	M	*KDM5B*	chr1:202,746,168	chr1:202,777,040	De novo	Dmiss	c.259A > T	p.Ile87Phe	Intellectual developmental disorder (AR)	LP
BRK-11–01	F	*KCNMA1*	chr10:78,647,208	chr10:76,887,450	De novo	frameshift	c.3526dupA	p.Met1176fs	Paroxysmal nonkinesigenic dyskinesia with or without generalized epilepsy (AD)	LP
BRK-86–01	M	*EIF5A*	chr17:7,214,392	chr17:7,311,073	De novo	Dmiss	c.221C > T	p.Pro74Leu	Faundes-Banka syndrome (AD)	LP
BRK-73–01	M	*MTOR*	chr1:11,217,287	chr1:11,157,230	De novo	Dmiss	c.4391A > G	p.Asp1464Gly	Smith-Kingsmore syndrome (AD)	LP
BRK-77–01	M	*GRIN2B*	chr12:13,769,471	chr12:13,616,537	De novo	Dmiss	c.1246 T > C	p.Phe416Leu	Intellectual developmental disorder with or without seizures (AD)	LP
sBRK-05–01	M	*MYO5A*	chr15:52,676,446	chr15:52,384,249	De novo	Dmiss	c.1826G > A	p.Arg609His	Griscelli syndrome, type 1 (AR)	LP
BRK-13–01,	M,	*DNM1*	chr9:130,982,567	chr9:128,220,288	Paternal	Dmiss	c.796C > T	p.Arg266Cys	Developmental and epileptic encephalopathy (AD)	VUS
BRK-13–04,	M,									
BRK-13–05	F									
**B. SNVs + indels (recessive)**										
**Proband-ID**	**Sex**	**Gene**	**Position (hg19)**	**Position (hg38)**	**Inheritance**	**Variant type**	**HGVS.c**	**HGVS.p**	**Relevant OMIM phenotypes**	**ACMG**
BRK-87–01	M	*NBN*	chr8:90,983,432	chr8:89,971,204	HOM	Dmiss	c.671G > A	p.Gly224Glu	Nijmegen breakage syndrome (AR)	VUS
BRK-88–01	F	*TRAPPC9*	chr8:141,370,230	chr8:140,360,131	HOM	Stop gain	c.1708C > T	p.Arg570*	Intellectual developmental disorder (AR)	P/LP
BRK-54–01	F	*TSEN2*	chr3:12,544,783	chr3:12,503,284	HOM	Dmiss	c.331G > A	p.Ala111Thr	Pontocerebellar hypoplasia type 2B (AR)	VUS
BRK-57–01	F	*UBR1*	chr15:43,347,084	chr15:43,054,886	HOM	Dmiss	c.1295 T > C	p.Ile432Thr	Johanson-Blizzard syndrome (AR)	VUS
BRK-58–01	M	*MED17*	chr11:93,523,767	chr11:93,790,601	HOM	Dmiss	c.445A > G	p.Lys149Glu	Microcephaly, postnatal progressive, with seizures and brain atrophy (AR)	VUS
BRK-59–01	M	*TIAM1*	chr21:32,513,486	chr21:31,141,168	HOM	Dmiss	c.3724G > A	p.Gly1242Arg	Neurodevelopmental disorder with language delay and seizures (AR)	VUS
BRK-74–01	M	*CTSA*	chr20:44,523,322	chr20:45,894,683	HOM	Dmiss	c.865G > C	p.Gly289Arg	Galactosialidosis (AR)	VUS
		*ZNF335*	chr20:44,578,150	chr20:45,949,511	HOM	Dmiss	c.3727G > T	p.Val1243Leu	Microcephaly (AR)	VUS
**C. SNVs + indels (X-linked)**										
**Proband-ID**	**Sex**	**Gene**	**Position (hg19)**	**Position (hg38)**	**Inheritance**	**Variant type**	**HGVS.c**	**HGVS.p**	**Relevant OMIM phenotypes**	**ACMG**
BRK-41–01	M	*PTCHD1*	chrX:23,353,126	chrX:23,335,009	X-linked	Dmiss	c.134G > A	p.Arg45His	Autism, susceptibility to, X-linked	VUS
BRK-43–01	M	*DMD*	chrX:32,663,098	chrX:32,644,981	X-linked	Dmiss	c.1132C > G	p.Gln378Glu	Duchenne muscular dystrophy, syndromic ASD	VUS
BRK-56–01	M	*SLC9A6*	chrX:135,080,654	chrX:135,998,495	X-linked	Dmiss	c.617G > A	p.Arg206Gln	Intellectual developmental disorder, X-linked	VUS
BRK-76–01	M	*WNK3*	chrX:54,278,075	chrX:54,251,642	X-linked	Dmiss	c.2413G > C	p.Glu805Gln	Intellectual disability	VUS
BRK-96–01	M	*WNK3*	chrX:54,337,648	chrX:54,311,215	X-linked	Dmiss	c.614A > G	p.Asn205Ser	Intellectual disability	VUS
**D. CNVs**										
**Proband_ID**	**Sex**	**Gene/s**	**Genomic disorder/cytoband**	**Type**	**Inheritance**	**Variant type**		**Size (kb)**	**Relevant phenotypes**	**ACMG**
BRK-81–01	M	Multiple	22q11.21	CNVs	De novo	Deletion		1400	Autism	P
BRK-65–01	M	Multiple	16p13.3	CNVs	Maternal	Duplication		1700	Autism	VUS
**E. TREs**										
**Proband_ID**	**Sex**	**Gene**	**Position (hg19)**	**Position (hg38)**	**Type**	**Inheritance**	**Variant type**	**Motif (TREs)**	**Relevant phenotypes**	
BRK-51–01	F	*SHANK2*	chr11:70,749,120–70749713	chr11:70,903,015–70903608	TREs	Dominant	intronic	AAAAT	Autism	
BRK-89–01	F	*NCOR2*	chr12:124,876,767–124,877,620	chr12:124,392,221–124,393,074	TREs	Dominant	intronic	ACAGAGAGACAGGGAGGAG;ACAGGGAGACAGGGAGGAG	-	
**F. mtDNA**										
**Proband-ID**	**Sex**	**Gene**	**Variant**	**Proband Heteroplasmy**	**Maternal Heteroplasmy**	**Disorder**				
BRK-16–01	M	MT-TL1	3243A > G	**2.1%**	Undetectable	MELAS				

*P* pathogenic, *LP* likely-pathogenic, *VUS* variant of uncertain significance

In addition to DN variants, we found recessive variants (homozygous) in 8 ASD/NDD genes (*TRAPPC9, NBN, TSEN2, UBR1, MED17, TIAM1, CTSA,* and *ZNF335*) in 7 families. All of which were Dmiss variants except for one stop-gain (p.Arg570* in *TRAPPC9*). Out of the 7 families with recessive events, 6 were consanguineous families (85.7%). Manual curation, according to ACMG guidelines, classified all recessive variants as VUS except for the *TRAPPC9* variant (p.Arg570*), which was already reported in ClinVar as pathogenic/likely-pathogenic. In addition, we identified five X-linked Dmiss variants in four genes (*PTCHD1*, *DMD, WNK3*, and *SLC9A6*) in 5 males with ASD, all of which were scored as VUS (Table 2).

#### Structural variants (CNVs + TREs)

Given the known association of ASD with genomic disorder regions, we investigated the overlap of CNVs detected within our patients with a list of regions where deletions and duplications were previously identified in individuals with ASD [12] (see “Methods”). We found two candidate variants: a de novo 1.4 Mb deletion in 22q11.21 and a 1.7 Mb maternally inherited duplication in 16p13.3 (Table 2). No other CNVs overlapped the known ASD/NDD gene list from our cohort. We further investigated TREs in known ASD genes [8] and found two matching TREs in *SHANK2* and *NCOR2* in two families (Table 2).

#### Mitochondrial variants

We investigated pathogenic mtDNA variants and heteroplasmy (where mutated mtDNA co-exist with unmutated mtDNA) that overlap previously reported variants (*n* = 15) associated with ASD [12]. We identified only one de novo variant (heteroplasmy of 2.1%) of the m.3243A > G variant associated with mitochondrial encephalopathy, lactic acidosis, and stroke-like episodes (MELAS) in an individual with ASD (maternal heteroplasmy was undetectable) (Table 2). We also considered overlap with mtDNA variants causing homoplasmic disorders generally affecting vision and hearing (*n* = 6) and found two matches: one individual with ASD had a 2.3% load of 14484 T > C variant (maternal genotype was undetectable), and a father had a 59.2% load of 11778G > A variant. Both variants are associated with Leber Hereditary Optic Neuropathy (LHON) syndrome (Additional file 1: Table S3).

Altogether, 27 families (27%) had at least one damaging variant in a known ASD/NDD gene panel in this cohort.

### Pathogenic variants in novel ASD-risk genes and regions

#### Small variants (SNVs + indels)

Beyond known genes, we searched genome-wide for damaging DN and homozygous variants (LoF, Dmiss) in novel candidate genes that could explain ASD in the remaining families.

For DN variants, we found 17 in as many genes (*CHD9, STAB2, MOV10, HDAC7, DNAJC10, SYNE3, COPS5, B4GALT1, DCAF17, FCHO2, INCENP, ING5, PTOV1, PRRC2C, TLN1, RRN3,* and *STRIP2)* in 14 families. Four were predicted LoF, all in genes, with pLI > 0.99 (*MOV10, HDAC7, TLN1,* and *CHD9)* and 13 were Dmiss variants. Three families had two damaging DN variants in two different genes each. All damaging DN variants in novel genes had additional carriers from ASD cohorts (MSSNG, SSC, and SPARK) (Additional file 1: Table S4).

We also looked for damaging homozygous variants (LoF and Dmiss) in genes with high pRec scores (> 0.9). Six novel genes (*TRIM29, EIF2A, CDH23, NOC3L, KDM8*, and *IFT140*) were identified in four families; five of which were affected by Dmiss variants and one by a LoF (splice acceptor variant, c.3236-1G > A) in *CDH23* (Table 3). Three of the four families with homozygous variants (75%), were consanguineous. We found additional biallelic variant carriers in ASD cohorts (MSSNG and SSC) for *CDH23* and *IFT140*.

**Table 3:** Recessive variants in novel candidate ASD/NDD genes/regions

**A. SNVs**											
**Proband-ID**	**Sex**	**Gene Name**	**Position (hg19)**	**Position (hg38)**	**Inheritance**	**Variant type**	**HGVS.c**	**HGVS.p**	**pRec score**	**Consanguinity**	**Additional carriers from MSSNG/SSC**
BRK-83–01	M	*EIF2A*	chr3:150,289,870	chr3:150,572,083	Homozygous	DMiss	c.937G > A	p.Gly313Arg	0.96462	Yes	0
		*TRIM29*	chr11:119,983,129	chr11:120,112,421	Homozygous	DMiss	c.1760C > T	p.Ala587Val	0.91016		0
BRK-23–01	M	*CDH23*	chr10:73,472,421	chr10:71,712,664	Homozygous	splice acceptor	c.3236-1G > A		1	No	1
BRK-61–01	F	*KDM8*	chr16:27,231,905	chr16:27,220,584	Homozygous	DMiss	c.1219G > T	p.Asp407Tyr	0.98296	Yes	0
		*NOC3L*	chr10:96,093,965	chr10:94,334,208	Homozygous	DMiss	c.2372 T > C	p.Phe791Ser	0.95724		0
BRK-71–01	M	*IFT140*	chr16:1,574,564	chr16:1,524,563	Homozygous	DMiss	c.3130C > T	p.Arg1044Cys	0.90704	Yes	1
**B. CNVs**											
**Proband-ID**	**Sex**	**Gene/s**	**Position (hg19)**	**Position (hg38)**	**Inheritance**	**Variant type**	**Location**	**Size (kb)**	**pRec score**	**Consanguinity**	**Additional carriers from MSSNG/SSC**
BRK-16–01	M	*AFG3L1P*	chr16:90,061,279–90061925	chr16:89,994,871–89,995,517	Homozygous	Deletion	Exonic	0.646	-	No	1
BRK-72–01	M	*ELOVL2*	chr6:10,978,990–10981316	chr6:10,978,757–10981083	Homozygous	Deletion	Exonic	2.326	0.15415	No	1
BRK-83–01	M	*FAM204A*	chr10:120,044,837–120057685	chr10:118,285,325–118,298,173	Homozygous	Deletion	Exonic	12.848	0.97978	Yes	3
BRK-12–01	M	*LINC00648/MIR548Y*	chr14:48,229,812–48,277,400	chr14:47,760,609–47808197	Homozygous	Deletion	Exonic	47.588	**-**	No	0

#### Structural variants (CNVs)

**Fig. 2:**
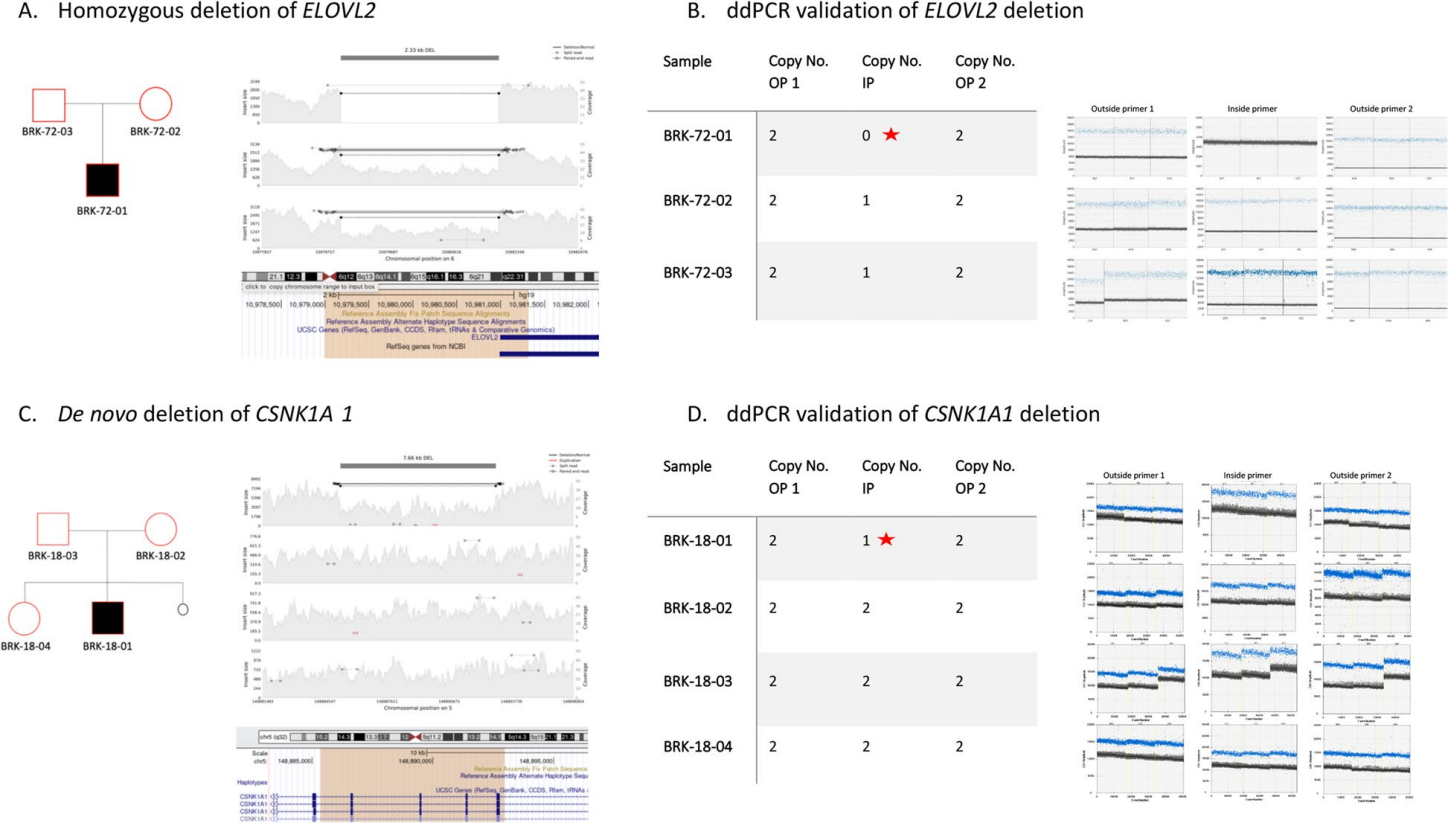
Examples of ASD-relevant CNVs. **A** Pedigree, IGV visualization, and UCSC genomic context of a 2.33 kb homozygous deletion comprising ~ 330 bp of exon 8 of *ELOVL2* (see colored region of the UCSC panel, http://genome.ucsc.edu). **B** ddPCR results showing a copy number of zero in the proband (indicated by red star), equivalent to no reads detected from the inside primer. **C** Pedigree, IGV visualization, and UCSC genomic context of 7.7 kb de novo deletion from a simplex family comprising exon 7 to 10 of CSNK1A1 gene (see colored region of the UCSC panel, http://genome.ucsc.edu). **D** ddPCR results showing copy number calculation equals to one in proband, heterozygous status, (indicated by red star) equivalent to less reads detected from inside primer in the proband sample. OP1 outside primer 1, OP2 outside primer 2, IP inside primer

A total of 5 ASD-associated CNVs were identified in 5 families. One was a de novo 7.7 kb deletion of exons 7 to 10 of *CSNK1A1* (Fig. 2, Additional file 1: Table S4). The other four were homozygous deletions in four families (Table 3) as follows: a 2.33 kb deletion in *ELOVL2* partially deleting exon 8 (Fig. 2), a 12.9 kb deletion overlapping exon 9 of *FAM204A*, a partial deletion of exon 11 (65 bp) in *AFG3L1P*, and a 47.6 kb deletion of full length long non-coding RNA gene (*LINC00648*) and complete deletion of a microRNA (*MIR548Y*). Most of these genes were novel in their association with ASD except for *ELOVL2*, which is reported in the SFARI Gene database (score 2). We checked if CNVs in these genes were found in additional individuals in global ASD cohorts and found a 6 kb deletion in *ELOVL2* in one family, a large de novo deletion (> 4 Mb) including *CSNK1A1* gene in one family*,* multiple large CNVs in six individuals that include *AFG3L1P* gene, and three individuals with deletions (> 12 kb) in *FAM204A*.

Altogether, we identify 28 candidate novel genes in 22 families (22%), of which 23 genes (82.1%) are supported by additional carriers in MSSNG and SSC, affected by variants in similar classes and zygosity.

## Discussion

The past decade has seen rapid advances in the discovery of genetic and genomic variants underlying complex neurodevelopmental conditions, including ASD [10, 11, 15, 61, 62]. Recently, WGS has emerged as a comprehensive approach for genomic discovery, enabling the detection of pathogenic variants spanning all types and size classes, including SNVs, indels, CNVs,TREs, and mtDNA [12, 63]. In this study, we present a comprehensive evaluation of genetic risk factors detected by WGS in a cohort of 100 families with ASD from vastly under-represented Middle Eastern populations as part of the first release of the BARAKA-Qatar Study.

**Fig. 3:**
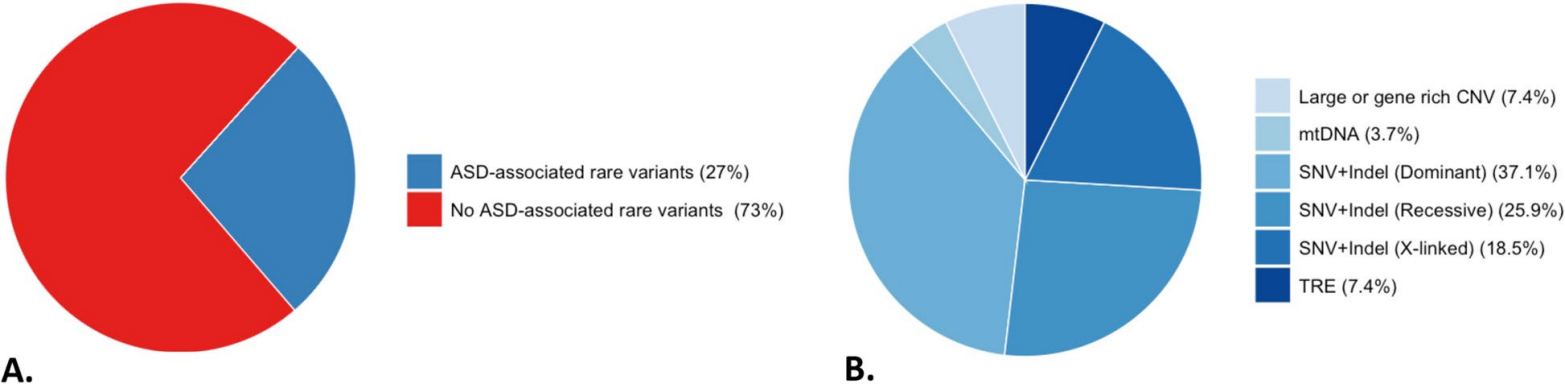
Genetic risk variants in known ASD/NDD genes. **A** Percentage ASD probands having candidate causative rare variants, stratified by **B** type of variant

We discover at least one candidate pathogenic variant in known ASD/NDD genes/regions in 27 families (27%) (Fig. 3A). Despite the high heritability of ASD, the majority of previously identified genetic risk appears to be from de novo variation [11]. Our cohort identified dominant risk variants, including de novo and inherited variants, in 15 of 27 (55.6%) families (37.1% de novo and 18.5% inherited). In terms of variant classes, the majority of dominant risk factor was from SNVs/indels (66.7%), followed by CNVs (13.3%), TREs (13.3%), and mtDNA variants (6.7%).

Notably, only two de novo SNVs (22.2%) were identified previously (p.Arg373Gln in *STAG1* and p.Ala1773Val in *SCN2A*) underscoring the high allelic heterogeneity underlying ASD across global populations. We sought to manually curate novel alleles according to ACMG criteria and found that all de novo novel alleles were classified as likely pathogenic. These mainly included damaging missense variants and only one frameshift variant (p.Met1176fs) in *KCNMA1*. *KCNMA1* encodes for potassium calcium-activated channel subfamily M Alpha 1 which are large conductance, voltage, and calcium-sensitive potassium channels fundamental to several physiological processes including smooth muscle contraction, neurotransmitter release, and neuronal excitability [64]. Mutations in this gene have been associated with a broad spectrum of neurological phenotypes and developmental disorders including cerebellar atrophy, DD, and seizures. A recent study reported *KCNMA1* mutations in individuals with ASD [64].

**Fig. 4:**
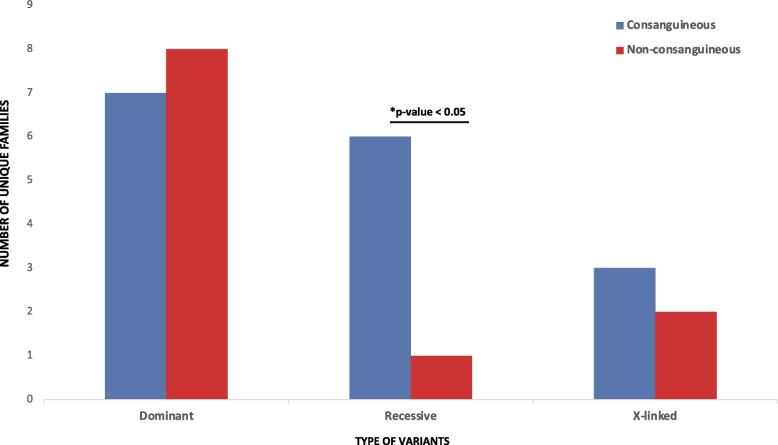
Genetic variants in known ASD/NDD genes stratified by consanguinity status of families. Recessive burden was significantly higher (*p*-value = 0.02) in consanguineous families

One of the most distinguishing features of middle eastern populations is the high degrees of consanguinity. While public databases comprise mostly outbred individuals, the local population of Qatar, for example has consanguinity levels of > 54% [65], suggesting that recessive architecture may contribute to a sizeable fraction of ASD etiology in this population. There have only been a few studies today examining ASD in consanguineous settings. One looked only at homozygous deletions and reported seven exonic deletions from 123 consanguineous families (5.7%) [66]. A more recent study investigating biallelic SNVs in highly consanguineous families found recessive gene risk in known ASD/NDD genes in 9 out of 23 (39%) families [17]. Data from our study suggest a recessive burden somewhere in between (6 of 44 consanguineous families (13.6%)). This burden is almost sixfold higher than in non-consanguineous families in our cohort, where only 1 of 56 families (1.8%) had a candidate homozygous causative variant in a known ASD/NDD gene (*p* = 0.02) (Fig. 4).

Moreover, in comparison to the largest WGS study investigating > 7,000 families with ASD from MSSNG and SSC cohorts which represent largely outbred populations [12], recessive genetic risk, accounting for different criteria used to define recessive events (i.e., recessive events with only LoF on both alleles were included in MSSNG/SSC), contributed to higher risk in our cohort (1.1%, 0.3%, and 3.7% for MSSNG, SSC, and BARAKA, respectively). Although the sample size of the BARAKA cohort is relatively modest at this time, these results highlight the potential impact of recessive variants on the etiology of ASD in highly consanguineous populations.

In some cases, the high levels of consanguinity may lead to certain challenges not anticipated when studying largely outbred cohorts. Among these are examples where it may be difficult to differentiate between driver and passenger mutations on a given haplotype. For example, two homozygous putatively damaging missenses variants (p.Gly289Arg, p.Val1243Leu) were identified in, two known NDD genes, *CTSA* and *ZNF335* a (neighboring genes on Chr 20) in a consanguineous male individual with ASD and ID. *ZNF335* plays an essential role in neurogenesis and biallelic variants in *ZNF335* have been associated with ASD-like phenotypes [67]. *CTSA* has been associated with an autosomal recessive form of Galactosialidosis (OMIM: 613111), for which intellectual disability is a common symptom.

We only had two multiplex families in the setting of parental consanguinity in our cohort (each with three affected siblings). While we expected to find recessive variants in these families, no candidate gene emerged with shared recessive variants across the three siblings. Instead, in one family (Family BRK-13), we found a paternally inherited heterozygous damaging missense variant (p.Arg266Cys) in *DNM1* that segregated with all affected siblings. A re-evaluation of the father's phenotype showed a diagnosis of ADHD and features of ASD. *DNM1* encodes dynamin 1, a GTP-binding protein mainly expressed in the central nervous system [68]. Pathogenic *DNM1* variants affect brain development and function and cause epileptic encephalopathy associated with global DD [69, 70]. Pathogenic variants in *DNM1* have also been reported in association with other clinical phenotypes such as hypotonia, movement disorder, ASD, cortical visual impairment, and microcephaly [69, 70]. The three affected siblings lacked epilepsy and showed symptoms of ADHD, although *DNM1* has not yet been associated with ADHD. Such an example of a multiplex family highlights the importance of taking a comprehensive approach with variant identification in each family, regardless of consanguinity status.

In addition, X-linked recessive variants (Dmiss) were found in 5 of 27 families (18.5%), supporting the role of the X-chromosome in ASD susceptibility in males. Variants in the X chromosome greatly contributed to ID and ASD in males with more than 140 genes being involved [62]. Two damaging missense variants (p.Glu805Gln and p.Asn205Ser) in *WNK3* were identified in two unrelated male probands. *WNK3* encodes a cell volume-sensitive kinase that is highly expressed during early brain development [71]. Previously, multiple hemizygous, LoF, and pathogenic missense variants were identified in *WNK3* in male individuals with sporadic and familial forms of ID [72]. Re-examination of comorbidities in the two probands in our cohorts reveals that neither had ID, and only one had ADHD, potentially representing an expansion of the *WNK3-*related phenotype.

In total, SNVs and Indels alone were present in 81.5% of our cohort, suggesting other variant classes could explain the missing heritability in the remaining families. Indeed, we employed WGS to enable the detection of CNVs and TREs associated with ASD. Our sample size was underpowered to detect significant enrichment of TREs in individuals with ASD compared to siblings without ASD. Only two families (7.4%) had TREs impacting known ASD genes. One of these was a (high functioning) female proband (Family BRK-51) with a TREs affecting intron 7 of *SHANK2, a* member of a family of scaffold proteins (comprising SHANK1, 2 and 3) that localize to the postsynaptic site of excitatory synapses in the central nervous system [73]. *SHANK2* has been implicated in various brain disorders, including ASD, ID, DD, ADHD, schizophrenia, epilepsy, and obsessive–compulsive disorder [74]. Another female proband (Family BRK-89) was diagnosed with Down syndrome disintegrative disorder (DSDD) (a developmental regression that leads to loss of previously acquired cognitive and social functioning, and the development of features of ASD) [75]. The genetic implications of DSDD have not yet been associated with any gene. We identified a TREs affecting intron 18 of *NCOR2*, a nuclear receptor corepressor 2 as part of a multi-protein corepressor complex known as the NCOR complex [76]. The NCOR complex plays a vital role in neurocognition with implications for autism [77].

Altogether, SNVs/Indels were the major risks affecting 22 of 27 families (81.5%: dominant (45.5%), recessive (31.8%), and X-linked (22.7%)) compared to CNVs (7.4%), TREs (7.4%), and mtDNA variant (3.7%) (Fig. 3B).

As only 27% of families had genetic risk from known ASD/NDD genes/regions, we expanded our search genome-wide for putatively novel genes or regions that could contribute to the genetic risk of ASD in the remaining families. Using similarly strict criteria as with known genes but limiting only to damaging de novo or homozygous variants, we identified candidate genes in 22 of 100 families (22%), 15 (68.2%) with de novo variants (SNVs 63.6%; CNVs 4.6%), and 7 families (31.8%) with homozygous variants (SNVs 13.6%, CNVs 13.6%, and one family (Family BRK-83) with both SNV and CNV (4.6%)). Of these novel genes, 23 out of 28 (82.1%) genes are supported by additional carriers affected by variants in similar classes and zygosity in ASD cohorts MSSNG, SSC, and SPARK. A further functional investigation is needed to determine the potential role of these Novel identified genes in ASD risk.

Notably, two families had multiple variants of the same type in known and novel genes, showing that finding a damaging variant in a known gene should not rule out searching for novel genes in the same family. First, the proband in (Family BRK-05) had a de novo Dmiss (p.Arg609His) in *MYO5A* (known gene, Table 2) and a de novo Dmiss (p.Asp428Gly) in *DCAF17* (Novel gene, Table S4). Second, proband in (Family BRK-48) had a de novo Dmiss (p.Tyr126Phe) in *WDR37* (known gene, Table 2) and a de novo Dmiss (p.Cys218Arg) in *COPS5* (Novel gene, Table S4). The high level of genotype/phenotype heterogeneity in individuals with ASD may explain the multiple variants/genes that could collectively contribute to the genetic risk of ASD. Comprehensive searches of known and novel genes contributing to ASD in each family help to determine the total burden of the disorder.

The use of WGS at point of care for families with ASD is relatively new in Qatar, where the public understanding of research as opposed to clinical testing still in its early stages. Genetic consultation is offered to individuals with significant genetic findings (i.e., variants classified as pathogenic or likely-pathogenic) to explain basic aspects such as recurrent risk based on mode of inheritance (de novo versus inherited) and interpretation of results. While study begins to set the scene for the integration of research findings into clinical practice, it nevertheless has important limitations which must be considered. First, our study sample size of 100 families limits generalizations at present about the relationship between consanguinity and ASD. While we observe an enrichment in recessive inheritance in such families, larger numbers will be needed to confirm if this trend will hold. Indeed, the BARAKA study has recently surpassed 250 families enrolled, with an eventual aim of 1000 families by end of 2024. As the cohort size increases, in particular from the local population where consanguinity exceeds 50%, we shall have valuable additional data to investigate this. Moreover, larger cohort sizes will allow us to move away from a per-family pathogenic variant approach to a cohort-level approach, using tools such as rare variant burden analysis [78] and/or gene-set enrichment analysis, which may aid novel gene discovery and uncover new ASD-implicated biological pathways. Similarly, larger datasets could be valuable in case–control studies that produce GWAS-like summary statistics, which can then support explorations of polygenic risk in ASD; such an effort is currently undermined in the absence of summary statistics from individuals with similar ancestry. Finally, combining our growing data with MSSNG in coming releases will make data from this unique ancestry available to global research endeavors which can then investigate more fully the genetic architecture in this part of the world compared to largely outbred populations.

## Conclusions

Taken as a whole, our study provides several important takeaways related to ASD research, especially in under-studied global populations. First, comprehensive characterization by WGS is a viable approach to identify genetic etiology in a substantial fraction of affected individuals. Second, we demonstrate the critical role played by de novo variants even in settings of high consanguinity, and thus the importance of enrolling parents where available to identify DNs with high specificity. Third, we observe a fourfold enrichment of homozygous causes in consanguineous families compared to non-consanguineous families; however, even in consanguineous and multiplex settings, the causative variant may be dominant/de novo, highlighting the necessity of comprehensively examining all variant classes before concluding a case study. Fourth, despite our cohort’s relatively high diagnostic yield, over 73% of families remain unresolved. The missing genetic risk could be due to common variants, rare variants in novel genes, variants in non-coding and regulatory regions, variants that could have been overlooked by subsequent prioritization and definition of damaging variants, or compound heterozygotes resulting from a combination of different variant classes (e.g., CNV on one allele and SNVs/indels on another). Accounting of these types of variants in the next release of the study may lead to genetic diagnosis in unresolved families. In all, we believe the BARAKA-Qatar study’s plans to continue growing cohorts with higher representation from the Middle East, North Africa and South Asia will help advance global understanding of ASD etiology in this region of the world.

### Supplementary Information

**Additional file 1:** **Table S1.** Detail phenotypes of individuals with ASD, **Table S2.** Known ASD/NDD gene panels, **Table S3.** Known mtDNA pathogenic variants associated with hearing loss/Deafness at greater than 2% heteroplasmy, **Table S4.** Candidate dominant variants in Novel genes/regions associated with ASD.

**Additional file 2:** **Figure S1.** Co-morbidities of Autism. Frequency of co-occurrence of phenotypes in individuals with ASD, **Figure S2.** Pairwise relationship of individuals. Related parents are in blue and above the threshold of kinship (>0.044) (dashed red line), **Figure S3.** Inbreeding coefficient (F). Per sample estimate of inbreeding of all individuals included. Individuals from inbred families are in blue, **Figure S4.** Sanger Sequencing. Validation of de novo variants.

## Data Availability

All data used and generated is available within the main manuscript and supporting files. The WGS data of families in this study have been submitted to MSSNG project and should be included in the coming DB7 release. Access to the data can be requested by completing data access agreement at https://research.mss.ng.

## Abbreviations

ASD: Autism spectrum disorder
BARAKA: Building a Resource for the Advancement of Knowledge of Autism
WGS: Whole genome sequencing
SNVs: Single nucleotide variants
CNVs: Copy number variants
SVs: Structural variants
TREs: Tandem repeats expansions
mtDNA: Mitochondrial DNA
ID: Intellectual disability
ADHD: Attention-deficit hyperactivity disorder
DD: Developmental delay
GI: Gastrointestinal
TADA: Transmission and de novo association
DN: *De novo*
LoF: Loss of function
Dmiss: Damaging missense
ACMG: American College of Medical Genetics
P: Pathogenic
LP: Likely-pathogenic
VUS: Variant of uncertain significance
QGP: Qatar-genome project
OMIM: Online Mendelian Inheritance in Man
SFARI: Simons Foundation Autism Research Initiative
MAF: Minor allele frequencies
