# Supplementary material for: Genomic architecture of autism spectrum disorder in Qatar: The BARAKA-Qatar Study
Source: Genome Med. 2023 Oct 7;15:81. doi: 10.1186/s13073-023-01228-w (PMC10560429; doi:10.1186/s13073-023-01228-w)
Supplement: Supplementary file 2 — Additional file 2: Figure S1. Co-morbidities of Autism. Frequency of co-occurrence of phenotypes in individuals with ASD, Figure S2. Pairwise relationship of individuals. Related parents are in blue and above the threshold of kinship (>0.044) (dashed red line), Figure S3. Inbreeding coefficient (F). Per sample estimate of inbreeding of all individuals included. Individuals from inbred families are in blue, Figure S4. Sanger Sequencing. Validation of de novo variants. [file 13073_2023_1228_MOESM2_ESM.docx]

**Figure S1:** **Co-morbidities of Autism**. Frequency of co-occurrence of phenotypes in individuals with ASD


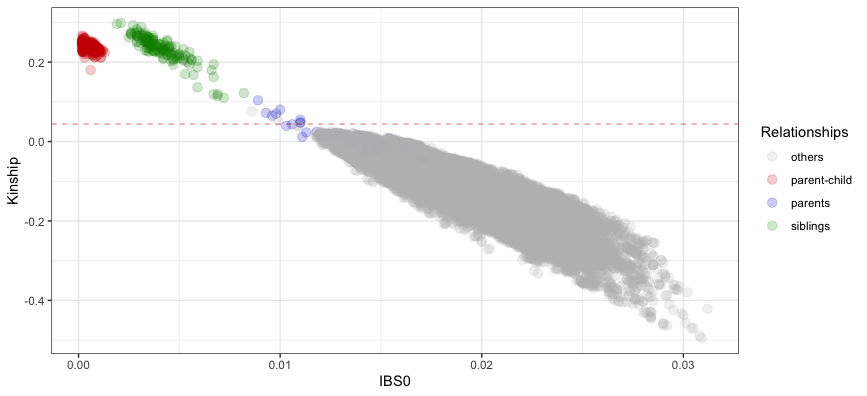


**Figure S2: Pairwise relationship of individuals.** Related parents are in blue and above the threshold of kinship (>0.044) (dashed red line).


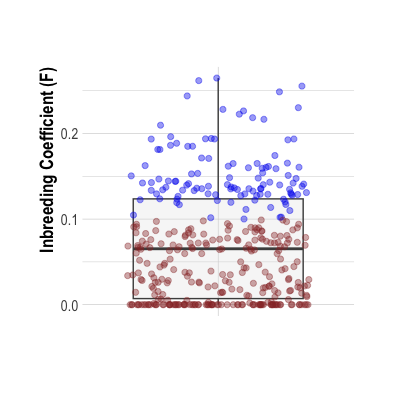


**Figure S3: Inbreeding coefficient (F).** Per sample estimate of inbreeding of all individuals included. Individuals from inbred families are in blue.


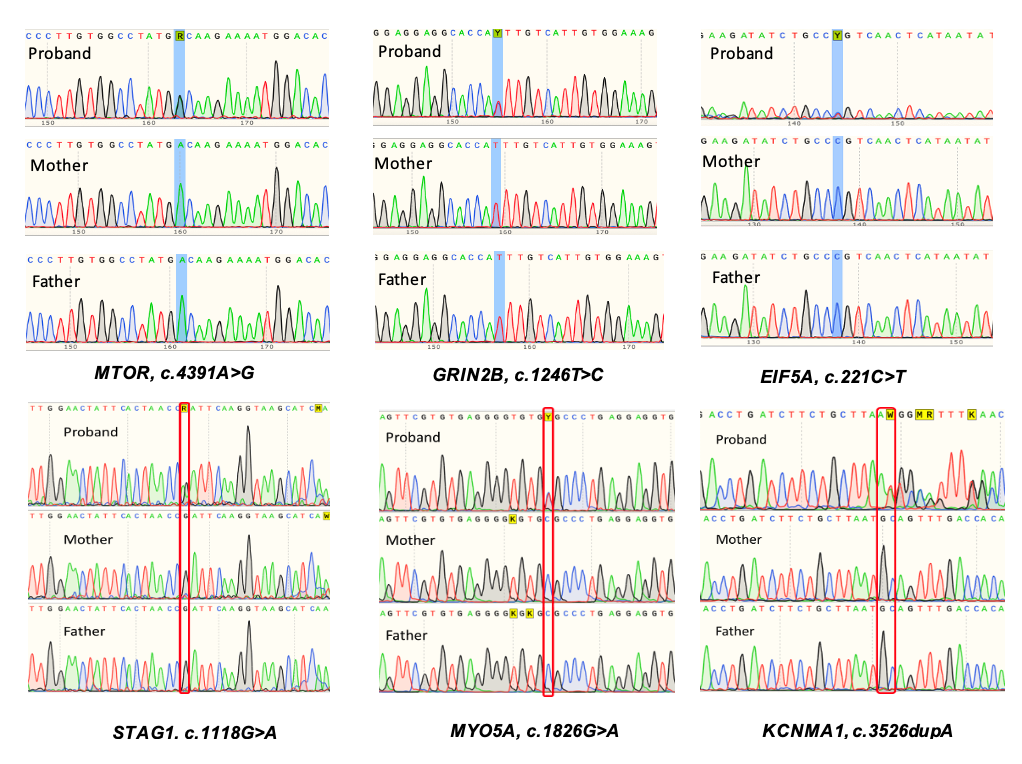


**Figure S4: Sanger Sequencing.** Validation of de novo variants
